# Increased DHA Production in Seed Oil Using a Selective Lysophosphatidic Acid Acyltransferase

**DOI:** 10.3389/fpls.2018.01234

**Published:** 2018-08-22

**Authors:** Pushkar Shrestha, Dawar Hussain, Roger J. Mulder, Matthew C. Taylor, Surinder P. Singh, James R. Petrie, Xue-Rong Zhou

**Affiliations:** ^1^Agriculture and Food, Commonwealth Scientific and Industrial Research Organisation, Canberra, ACT, Australia; ^2^Manufacturing, Commonwealth Scientific and Industrial Research Organisation, Clayton, VIC, Australia; ^3^Land and Water, Commonwealth Scientific and Industrial Research Organisation, Canberra, ACT, Australia

**Keywords:** lysophosphatidic acid acyltransferase, long chain polyunsaturated fatty acid, DHA, positional distribution, *sn*-2 enrichment

## Abstract

Metabolic engineering of the omega-3 (ω3) long chain polyunsaturated fatty acid biosynthesis pathway has generated fish oil-like levels of pharmaceutically and nutritionally important docosahexaenoic acid (DHA) in plant seeds. However, the majority of DHA has been accumulated at the *sn*-1 and *sn*-3 positions of triacylglycerol (TAG) in these engineered seeds, leaving only a minor amount (∼10%) at *sn*-2 position and indicating a strong discrimination (or, a very poor specificity) for DHA by seed lysophosphatidic acid acyltransferases (LPAATs), which mediate the acylation of *sn*-2 position of glycerol backbone. In order to increase the level of DHA at *sn*-2 position of TAG and to increase overall DHA level in seeds, we attempted to discover DHA-preferring LPAATs. Several LPAATs for acylation of the *sn*-2 position of the TAG glycerol backbone were investigated for substrate preference for DHA. In transiently expressing these LPAATs in *Nicotiana benthamiana*, a *Mortierella alpina* LPAAT had the highest substrate specificity for accumulating DHA onto oleoyl-lysophosphatidic acid (oleoyl-LPA), while the plant LPAATs tested showed lower preference for DHA. In a competition assay with a pool of four ω3 acyl-Coenzyme A (CoA) substrates involved in the DHA biosynthesis pathway, LPAATs from both *M. alpina* and *Emiliania huxleyi* showed a high preference for DHA-CoA acylation onto oleoyl-LPA. When docosahexaenoyl-LPA was used as the acyl receiver, *M. alpina* LPAAT also showed a high preference for DHA-CoA. Stable overexpression of *M. alpina* LPAAT in an Arabidopsis line that expressed the DHA biosynthesis pathway significantly increased both the total DHA levels and the distribution of DHA onto the *sn*-2 position of seed TAG. LC-MS analysis of the seed TAG species also confirmed that overexpression of *M. alpina* LPAAT increased di-DHA and tri-DHA TAGs, suggesting that the *M. alpina* LPAAT could enrich DHA at the TAG *sn*-2 position, leading to a metabolic engineering of oil seed for channeling DHA into the *sn*-2 position of TAG and to a higher DHA level.

## Introduction

Omega-3 long chain polyunsaturated fatty acids (ω3 LC-PUFAs), such as EPA (20:5ω3), DPA (22:5ω3), and DHA (22:6ω3) are important essential fatty acids for human health. Several investigations have indicated their health benefits, which range from their positive roles in fetal development through to osteoporosis, as well as preventing cardiovascular disease, diabetes, and Alzheimer’s disease (Swanson et al., 2012; Haslam et al., 2013). The demand for these fatty acids is increasing for dietary purposes, medical treatments, and livestock and aquaculture feed. The main source of fatty acids, marine fish stock, is not increasing, however, and the demand is now exceeding global supplies of fish oil (Tacon and Metian, 2009). To create an alternative, sustainable and cost-effective source, several metabolic engineering efforts have been trialed to synthesize ω3 LC-PUFA in seed oil. Currently, there have been few successes in the production of moderate to high levels of ω3 LC-PUFA in seeds. For example, Cheng et al. (2010) reported the production of 25% EPA in *Brassica carinata* seeds. More recently, we have achieved the production of 15% DHA in Arabidopsis seed oil (Petrie et al., 2012) and 12% DHA in *Camelina sativa* seed oil (Petrie et al., 2014) by expression of seven transgenes for the DHA synthesis pathway under the control of seed-specific promoters. The majority of DHA is located at the *sn*-1 and *sn*-3 positions (∼90%) of TAG, with only a small proportion found at the *sn*-2 position (∼10%) (Petrie et al., 2012, 2014).

There has been some debate about the relative nutritional benefits of LC-PUFA accumulated at the *sn*-2 or *sn*-1/*sn*-3 positions of TAG for dietary intake. The distribution of ω3 LC-PUFAs at the *sn*-1 and *sn*-3 positions dominates the TAG in seal oil (Wanasundara and Shahidi, 1997), but in fish oil the *sn*-2 position is dominant. During TAG digestion, pancreatic lipase preferentially cleaves fatty acids from the TAG *sn*-1 and -3 positions in the intestine, resulting in free fatty acids and *sn*-2 MAG (Yang and Kuksis, 1991). Long chain fatty acids are absorbed in intestinal enterocytes by a slow protein-mediated process. In contrast, *sn*-2 MAG is simply absorbed by passive diffusion and serves as a primary backbone for gut or liver phospholipid synthesis or reassembly of the TAG (Schulthess et al., 1994). Also, pancreatic lipase has shown lower specificities for ω3 LC-PUFA located at the *sn*-1 and -3 positions compared to medium chain fatty acids, resulting in slower hydrolysis of the TAG (Ikeda et al., 1995) and slower lymphatic absorption in rat trials (Christensen et al., 1994). Therefore, it might seem that LC-PUFA located at the TAG *sn*-2 may be more important for better bioavailability than that located at the *sn*-1 and *sn*-3 positions. There is also evidence of health benefits of seal oil, however, in which ω3 LC-PUFAs are distributed predominantly at the TAG *sn*-1 and *sn*-3 positions. For example, rat feeding experiments with seal oil or fish oil-rich fats that have equivalent polyunsaturated/monounsaturated/saturated fatty acids and ω6/ω3 PUFA ratios showed that seal oil more effectively reduced serum and liver TAG concentrations than fish oil (Yoshida et al., 1999).

Regardless of the respective nutritional benefits of ω3 LC-PUFA distribution at the *sn*-2 or *sn-*1/*sn*-3 positions of TAG, further preferential enrichment of DHA at the *sn*-2 position above current levels in the engineered Arabidopsis or camelina seed oil may ultimately increase the overall DHA production.

Lysophosphatidic acid acyltransferase (LPAAT) is responsible for acylation of the *sn*-2 position of glycerol backbones in the Kennedy pathway of TAG synthesis. LPAATs have been shown to have differential acyl preferences. For example, the LPAAT isolated from rapeseed (*Brassica napus* Reston) that accumulates high levels of the long chain unsaturated fatty acid, erucic acid (22:1) in TAG, had poor specificity for acylating 22:1 onto the *sn*-2 position of lysophosphatidic acid (LPA), while it showed preferences for C18 unsaturated acyl-CoA substrates (Sun et al., 1988; Bernerth and Frentzen, 1990; Cao et al., 1990). On the other hand, meadowfoam (*Limnanthes alba*) seed LPAAT showed a strong preference for 22:1 (Cao et al., 1990). Transgenic expression of the meadowfoam seed LPAAT in rapeseed significantly increased the level of 22:1 at the TAG *sn*-2 position from 0.3 to 15.1% of total fatty acid (Lassner et al., 1995). Our observation of the location of DHA in transgenic Arabidopsis and camelina seed oil suggested that the Arabidopsis and camelina seed LPAATs should have a lower preference for DHA.

Here, we have compared seven LPAAT enzymes from different organisms, with either known or suspected preference for acylating long-chain fatty acids, with those not expected to catalyze efficient DHA incorporation at the *sn*-2 position (as controls). More specifically, LPAAT2 from Arabidopsis (AtLPAAT2) is an endoplasmic reticulum-localized enzyme shown to have activity on C16 and C18 substrates, but its activity on C20 or C22 substrates has not been tested (Kim et al., 2005). LPAAT from meadowfoam (*L. alba*, LaLPAAT) has shown a preference for C22:1 (Lassner et al., 1995). The yeast LPAAT Slc1p (*Saccharomyces cerevisiae*, ScLPAAT) has been shown to have activity with 22:1-CoA in addition to 18:1-CoA substrates, indicating a broad substrate specificity with respect to chain length (Zou et al., 1997), although 22:6-CoA, 22:5-CoA, and other LC-PUFAs had not been tested as substrates. *Mortierella alpina* is a fungus producing a high level of arachidonic acid (20:4, ω6) (Bajpai et al., 1991). Expression of MaLPAAT in transgenic *Yarrowia lipolytica* has resulted in higher accumulation of EPA and DHA in total fatty acid (Patent US 7879591), but its activity in plant cells was unknown. *Micromonas pusilla* is a microalga that produces and accumulates DHA in its oil, although the positional distribution of the DHA on TAG in this species has not been confirmed (Dunstan et al., 1992). *E. huxleyi* is marine phytoplankton and is rich in EPA and DHA (Pond and Harris, 1996). The LPAAT from these organisms might prefer to LC-PUFA substrates.

Our previous results that the majority of DHA is located at the *sn*-1 and *sn*-3 positions of TAG in the engineered Arabidopsis or camelina (Petrie et al., 2012, 2014) led to the hypothesis that the Arabidopsis or camelina LPAAT might have low preference for DHA. *B. napus* is another member from the same Brassicaceae family as Arabidopsis or camelina. The preference of *B. napus* LPAAT (BnLPAAT, Maisonneuve et al., 2010) for DHA is also unknown. The LPAATs from the above organisms were thus chosen in this study to determine their preference for various ω3 LC-PUFAs in comparison to AtLPAAT and BnLPAAT. The activities were examined by transient expression in *Nicotiana benthamiana* leaves and biochemical analysis of microsomal proteins from these leaves. We found that *Mortierella alpina* LPAAT (MaLPAAT) had the highest preference for DHA among the tested LPAATs, in terms of both total and relative activity, while *Emiliania huxleyi* LPAAT (EhLPAAT) had the highest preference for DPA, but low total activity. Super-transformation of MaLPAAT into an Arabidopsis background previously engineered with the DHA biosynthesis pathway resulted in the enhanced distribution of DHA at the *sn-*2 position, as well as increased amounts of DHA in the seed.

## Materials and Methods

### Materials and Chemicals

Glycerol-3-phosphate (G3P) and [^14^C(U)]-glycerol-3-phosphate were purchased from Sigma and PerkinElmer. Acyl-CoAs were purchased from Avanti Polar Lipids Inc., or synthesized in our laboratory according to the method described (Sánchez et al., 1973). 18:1-LPA and 22:6-LPA were synthesized by acylation of unlabelled or labeled G3P adapted from the method described (Kanda and Wells, 1981). Trifluoroacetic acid and trifluoroacetic anhydride for LPA synthesis were purchased from Sigma. Synthesized LPA and PA in the mixture were separated by TLC on silica gel 60 plate (Merck), developed with chloroform/methanol/acetic acid/water (90/15/10/3, v/v/v/v). LPA band was recovered from TLC plate and quantified by GC or liquid scintillation counting.

### Gene Constructs and Transient Expression in Leaf

The LPAAT sequences from *Arabidopsis thaliana* (NP_567052), *B. napus* (NP_001302955), *S. cerevisiae* (NP_010231), *M. alpina* (AED33305), *M. pusilla* (XP_002501997), *E. huxleyi* (XP_005765016), *Limnanthes alba* (AAC49185) were synthesized at GeneArt (Thermo Fisher Scientific), codon optimized, and cloned into binary vector pJP3343 (Reynolds et al., 2015), under the control of the 35S promoter. The resulting constructs were transformed into *Agrobacterium tumefaciens* strain AGL1. Whole leaves of *N. benthamiana* were infiltrated together with a viral silencing protein p19 (Voinnet, 2003) essentially as described previously (Wood et al., 2009). Briefly, AGL1 carrying different expression plasmids were grown overnight at 28°C in LB broth containing appropriate antibiotics. Cells were spun down and resuspended into infiltration buffer (5 mM 4-morpholineethanesulfonic acid, 5 mM MgSO_4_, pH 5.7, 100 uM acetosyringone), and incubated for 3 h. Optical density of each culture were adjusted to final OD_600_ 0.3 before infiltration. Microsomal proteins from infiltrated leaves after 5 days were prepared according to previously described (Zhou et al., 2013).

### LPAAT *in vitro* Assay and Fatty Acid Analysis

Enzyme assays were carried out either using ^14^C labeled LPA substrates or non-radiolabelled substrates in 2 mL glass GC vials (Agilent Technologies). The enzyme assay consisted of 6 nmol [^14^C]-LPA, 6 nmol acyl-CoA, 50 μL assay buffer (0.2 M Tris-HCl pH 7.0, 0.4 M sucrose, 20 μg/μL essentially fatty acid-free BSA) and 100 μg microsomal proteins in a total volume of 100 μL. The LPA solution in 2% methanol/chloroform (v/v) was transferred to the glass vial and evaporated under nitrogen gas before adding other ingredients. The reaction was started by adding the microsomes, then incubating at 25°C for 15 min, with shaking at 700 rpm. The reaction was terminated by adding 1.2 mL chloroform/methanol (2/1, v/v) and 0.3 mL 0.1 M KCl and stored in ice. The mixture was agitated in a Vibramax (Heidolph) at 2500 rpm for 7 min, centrifuged at 1700 × *g* for 5 min and the lower lipid phase was collected. The solvent was evaporated from the lipids under a flow of nitrogen gas. [^14^C]-PA product was resolved by TLC (silica 60, MERCK) developed with chloroform/methanol/acetic acid/water (90/15/10/3, v/v/v/v) and the radiolabelled lipid spots visualized using a Phosphor Imager (Fujifilm). The amount of radiolabel (DPM count) in PA was counted with a Tri-Carb 2810TR liquid scintillation counter (PerkinElmer).

The substrate competition experiments with four acyl-CoA molecular species were carried out essentially as above with some modifications. The 150 μL reaction mixture contained 30 nmol 18:1- or 22:6-LPA, 15 nmol each of cold 18:3-, 20:5-, 22:5-, 22:6-CoAs, 75 μL the assay buffer as mentioned above and 150 μg microsomal protein. The mixture was incubated for 60 min at 25°C with shaking and the reaction terminated by adding 1.2 mL chloroform/methanol (2/1, v/v) and 0.25 mL 1% acetic acid in 0.1 M KCl. The lipids were extracted and the PA fractionated by TLC as mentioned above. For resolution and quantification of fatty acids, PA spots were transferred to glass vials spiked with known amounts of triheptadecanoin as internal standard (Nu-Chek Inc., United States). Fatty acid methyl esters (FAMEs) were prepared by incubating in 0.7 mL 1N methanolic-HCl (Supelco) for 2 h at 80°C. FAMEs were extracted after adding 0.9% NaCl and hexane, and analyzed by GC as described (Zhou et al., 2011) using 30-m BPX70 column. The ramping program was modified to an initial temperature at 150°C holding for 1 min, raised to 210°C at 3°C/min, then to 240°C at 50°C/min, finally holding for 1.4 min. FAMEs were quantified using ChemStation (Agilent).

### Stable Expression of MaLPAAT in Seed

Seed expression construct Fp1::MaLPAAT with *NptII* selection marker was prepared by replacing the 35S promoter in above 35S::MaLPAAT construct with *B. napus* seed specific *Fp1* promoter (Stalberg et al., 1993). The DHA-producing transgenic Arabidopsis line (GA7) (Petrie et al., 2012) was super-transformed by *Agrobacterium* mediated floral dip method for MaLPAAT. The super-transformed seeds (T_1_) were screened for kanamycin resistance by germination and growing seedlings on MS plates containing 40 mg/L kanamycin. Presence of *Pavlova salina* Δ4-desaturase gene from the GA7 parent, as well as *MaLPAAT* and *NptII* genes from the super-transformant was confirmed by PCR in leaf DNA of T_1_ plants. PCR primers were 5′-ATCTTCTAACCCTGTGCTCC (forward) and 5′-AGATCAGCCTTATCGAGCCT (reverse) for *MaLPAAT* gene, 5′-AGCCTACTGATGCTTGGTCA (forward) and 5′- CTTAACAAGAGGAGCAAGTCT (reverse) for *P. salina* Δ4-desaturase gene, or 5′- AAGATGGATTGCACGCAGGT (forward) and 5′-TGATGCTCTTCGTCCAGATC (reverse) for *NptII* gene. Mature T_2_ seeds from T_1_ plants were harvested and analyzed by GC for their fatty acid profiles as above. The DHA content at the *sn*-2 position of seed TAGs was analyzed as described below.

### Seed Fatty Acid Analysis

Fatty acid methyl esters were prepared by incubating seeds in 1N methanolic-HCl (Supelco) as above. The FAMEs were extracted after mixing and centrifugation of the mixture. FAMEs were analyzed by GC as described above.

### Positional Analysis of Fatty Acids in Seed Oil and Triacylglycerols

Triacylglycerols were purified from T_2_ seed oils and were used for positional analysis of fatty acids at *sn-*2 position of the TAG as described by Bafor et al. (1990) with some modifications. One mg of TAG in 5% gum arabic were added to the 200 μL reaction buffer containing 0.1 M Tris-HCl pH7.7, 5 mM CaCl_2_ and 25 U freshly added *Rhizopus arrhizus* lipase (Fluka). The mixture was incubated for 4 min at 30°C while mixing at 700 rpm. The reaction was terminated by adding 1.2 mL of chloroform/methanol (2/1, v/v) and 0.2 mL of 1M KCl. The mixture was agitated for 5 min and then centrifuged for 5 min. The lower lipid phase was transferred to a new vial. The upper phase was washed once again with 0.8 mL chloroform, and the lipid extracts were pooled. The solvent was evaporated from the lipid sample and dissolved in a small volume of chloroform. For *sn*-2 MAG fractionation, the lipid sample was loaded on a 2.3% boric acid-impregnated TLC (silica 60, MERCK) and developed with hexane/diethyl ether/acetic acid (50/50/1, v/v/v), alongside with authentic lipid standards (NuChek Inc). The undigested TAG was also run on the same plate. The lipid bands were viewed under UV after spraying 0.01% primuline in acetone/water (8/2, v/v). The TAG and *sn-*2 MAG bands were collected, and their FAMEs were prepared and analyzed by GC as above. Positional analysis of DHA in the purified TAG from T_4_ homozygous seeds was further investigated by ^13^C NMR analysis as described previously (Petrie et al., 2014).

### LC-MS Analysis of Seed Lipids

Total lipids extracted from 1 mg of T_4_ homozygous seeds were dissolved 1 mL of butanol/methanol (1/1, v/v) containing 10 mM butylated hydroxytoluene. DAG and TAG species that contained DHA were analyzed using MRM mode by LC-MS as previously described (Shrestha et al., 2016). The ratio of the peak area of individual species among total DHA-containing DAGs or TAGs was calculated.

## Results

### Transient Expression of LPAATs in *N. benthamiana* Leaves

**FIGURE 1:**
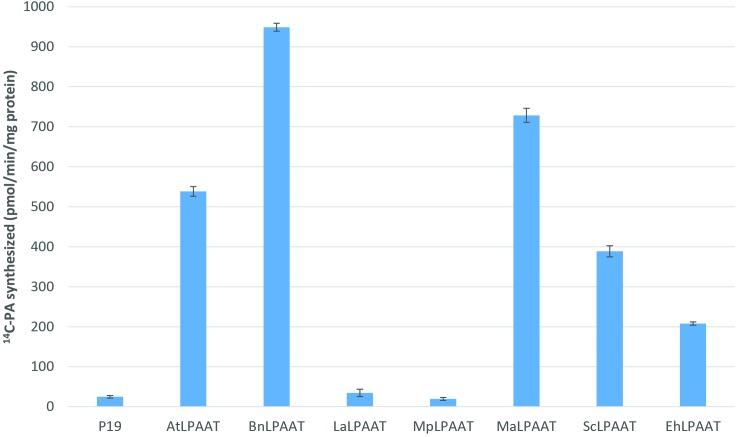
Enzymatic activities of various transiently expressed LPAATs on 18:3-CoA. LPAAT activities were determined by measuring the amounts of PA formed after incubating the substrates ^14^C-*sn*-1-18:1-LPA and 18:3-CoA with leaf microsomal membrane proteins expressing different LPAATs in the presence of p19 silencing suppressor protein. Activity from the p19 alone as control represents the leaf endogenous LPAAT activity. The error bars denote standard deviations of the means from triplicate assays.

We carried out *Agrobacterium*-mediated transient expression of seven individual transgenic LPAATs in the presence of the p19-viral gene silencing suppressor in *N. benthamiana* leaves. The expression of p19 alone was used as a control. The microsomal proteins from the leaves expressing exogenous LPAATs produced ^14^C-PA in the presence of ^14^C-*sn*-1-oleoyl-LPA and 18:3-CoA (**Figure 1**), indicating the LPAAT enzymes were active, and mediated transfer of the acyl group from acyl-CoA to the *sn*-2 position of *sn*-1-LPA. The LPAAT enzymes from *A. thaliana* (*At*), *B. napus* (*Bn*), *M. alpina* (*Ma*), *S. cerevisiae* (*Sc*), and *E. huxleyi* (*Eh*) showed greater LPAAT activity (538.3 ± 12.2, 948.7 ± 10.0, 728.4 ± 17.4, 388.7 ± 13.8, and 208.0 ± 3.9 pmol/min/mg protein, respectively). Compared with the p19 control (24.8 ± 3.1 pmol/min/mg), they were 22-, 38-, 29-, 16-, and 8-fold higher than the rate observed for the endogenous LPAAT activity, respectively. The LPAATs from *L. alba* (*La*) or *M. pusilla* (*Mp*), however, showed low levels of activity for 18:3-CoA substrate (34.7 ± 9.2 and 19.4 ± 3.7 pmol/min/mg protein, respectively).

### Preferences of LPAATs for LC-PUFA Acyl-CoA Species

**FIGURE 2:**
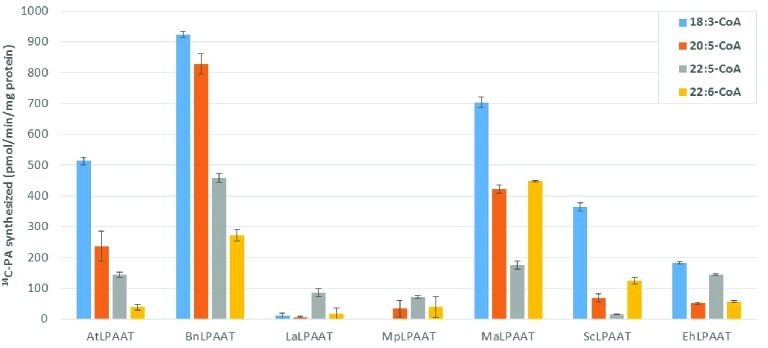
Substrate preferences of transiently expressed LPAATs in *N. benthamiana* for acyl-CoA donors. LPAAT activities on acyl-CoA donors with ^14^C-*sn*-1-18:1-LPA as acyl receiver. Error bars represent standard deviations of the means from triplicate assays.

We compared the preference of the LPAATs above for long chain polyunsaturated fatty acyl donors, using individual acyl-CoAs, namely 18:3-CoA, 20:5-CoA, 22:5-CoA, or 22:6-CoA, with ^14^C-*sn*-1-oleoyl-LPA as the acyl receiver in the assays. The enzyme activities were calculated based on the PA products formed, subtracting the endogenous LPAAT product (p19 control) from the same acyl-CoA donor, which were 24.8 ± 3.1, 9.2 ± 3.0, 11.2 ± 1.0, and 8.8 ± 4.0 pmol/min/mg microsomal protein for 18:3-CoA, 20:5-CoA, 22:5-CoA, or 22:6-CoA, respectively. AtLPAAT revealed a higher specific activity of 513.5 ± 12.2 pmol/min/mg protein for 18:3-CoA (**Figure 2**) and lower activities for 20:5-, 22:5-, and 22:6-CoA at 236.8 ± 49.5, 143.2 ± 9.1, and 39.4 ± 9.6 pmol/min/mg protein, respectively. BnLPAAT showed even higher levels of activity: 923.9 ± 10.0, 828.7 ± 32.2, 457.2 ± 13.7, and 272.3 ± 18.9 for 18:3-CoA, 20:5-CoA, 22:5-CoA, and 22:6-CoA, respectively. A common characteristic observed in these two LPAATs was that their substrate preferences decreased for longer-chain and more-unsaturated ω3-LC-PUFAs (C20-C22 LC-PUFAs). In contrast, both MaLPAAT and ScLPAAT displayed a greater preference for 22:6-CoA (447.7 ± 3.3 and 124.7 ± 10.4 pmol/min/mg microsomal protein, respectively) than 20:5-CoA (422.1 ± 14.2 and 69.1 ± 12.7 pmol/min/mg microsomal protein, respectively) and 22:5-CoA (174.9 ± 14.3 and 14.6 ± 1.3 pmol/min/mg microsomal protein), although all their activities were lower than when 18:3-CoA was used as the acyl donor (**Figure 2**). EhLPAAT showed an overall lower activity on different acyl-CoAs, with 183.2 ± 3.9, 50.8 ± 2.1, 144.3 ± 2.8, and 56.8 ± 3.0 pmol/min/mg microsomal protein for 18:3-, 20:5-, 22:5-, and 22:6-CoA, respectively. Both LaLPAAT and MpLPAAT showed very low activity, but with a preference for 22:5-CoA (85.9 ± 13.8, and 71.9 ± 5.0 pmol/min/mg microsomal protein, respectively), compared with 18:3-CoA (**Figure 2**).

It is of interest to compare the relative specificity of LPAATs for LC-PUFA vs. ALA because all the tested LPAATs with high activities favored 18:3-CoA. MaLPAAT and ScLPAAT had their highest relative activities with 22:6-CoA, at 63.6 ± 0.5% and 34.3 ± 2.9% of the activity relative to 18:3-CoA, respectively. AtLPAAT and BnLPAAT showed 7.7 ± 1.9% and 29.5 ± 2.0% of the activity with 22:6-CoA relative to 18:3-CoA, respectively. BnLPAAT and AtLPAAT showed higher relative specificity for other LC-PUFA-CoAs, however, such as 20:5-CoA and 22:5-CoA. BnLPAAT exhibited 89.7 ± 3.5% and 49.5 ± 1.5% relative specificities for 20:5-CoA and 22:5-CoA, compared with 18:3-CoA. EhLPAAT showed the highest relative specificity for 22:5-CoA (78.8 ± 1.5% of the activity relative to 18:3-CoA) among all tested LPAATs. Due to the overall low activities of LaLPAAT and MpLPAAT, their relative specificities for LC-PUFA vs. ALA are not shown.

### Competition Among Acyl Donor Substrates for LPAATs

Lysophosphatidic acid acyltransferases activity for an acyl-CoA might also depend on the competition of other acyl-CoA species in the *in vivo* acyl-CoA pool, where multiple acyl-CoAs are present. Therefore, we carried out acyl competition assays to estimate LPAAT activity for an acyl-CoA in the presence of multiple acyl-CoAs in a single reaction. In this experiment, we attempted to use four ω3 acyl-CoA substrates involved in the DHA biosynthesis pathway. Each reaction used *sn*-1-oleoyl-LPA (18:1-LPA) or *sn*-1-docosahexaenoyl-LPA (22:6-LPA) as the acyl acceptor, and equimolar amounts of 18:3-, 20:5-, 22:5- and 22:6-CoAs as acyl donors.

**FIGURE 3:**
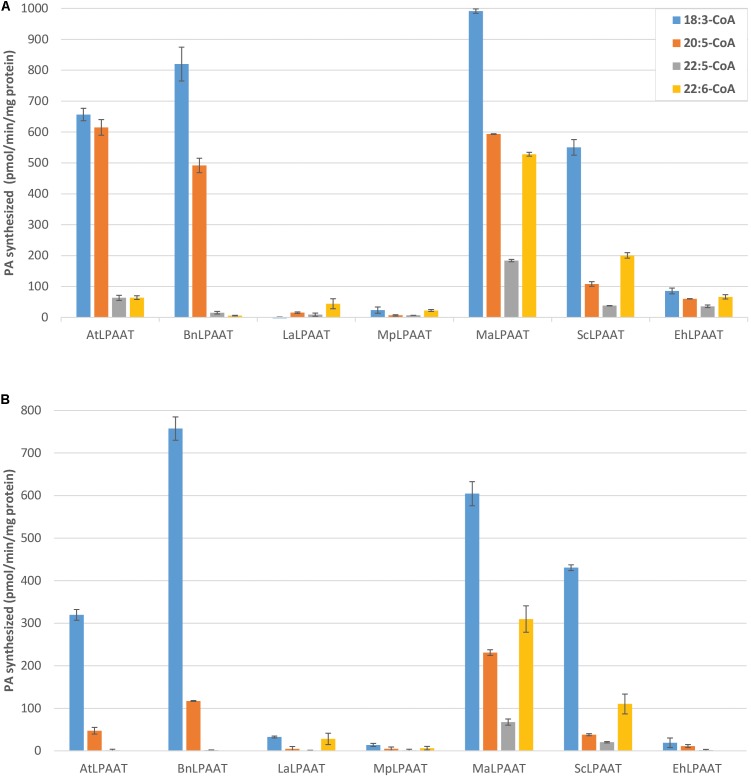
Competition for acyl-CoA substrates by LPAATs transiently expressed in *N. benthamiana*. LPAAT activities with 18:1-LPA **(A)** or 22:6-LPA **(B)** and 18:3-, 20:5-, 22:5-, or 22:6-CoA in the multiple acyl-CoAs supplied. Error bars represent standard deviations of the means from triplicate assays.

Several control assays were performed first, including microsomal protein provided with either LPA substrate alone, acyl-CoAs substrate alone, or no substrate. Results from all three control assays showed similar minimal levels of PA, indicating that the levels of PA contributed by the microsomal preparation or endogenous LPAAT activity were insignificant (data not shown). Nevertheless, for each LPAAT tested, the leaf endogenous LPAAT activity and the trace amount of PA in p19 control were subtracted from the total LPAAT activity for each acyl-CoA. Then, the relative activities of the LPAATs were calculated, by comparing the activities for each acyl-CoA to its activity for 18:3-CoA, which demonstrated the highest activity in all the LPAAT enzymes studied (**Figure 3A**). The microsomal proteins from p19-infiltrated leaf showed some basal LPAAT activities with the acyl-CoAs used (75.3 ± 4.5, 8.4 ± 7.2, 15.0 ± 1.1, and 7.8 ± 6.9 pmol/min/mg protein for 18:3-, 20:5-, 22:5-, and 22:6-CoAs). These values were subtracted from the activities observed in the microsomal proteins from LPAAT-infiltrated leaves. The specific activities of AtLPAAT, BnLPAAT, MaLPAAT, ScLPAAT, and EhLPAAT for 18:3-CoA were 656.6 ± 20.2, 820.0 ± 54.5, 991.4 ± 7.3, 550.3 ± 25.2, and 85.5 ± 9.3 pmol/min/mg protein, respectively.

AtLPAAT activity with 20:5-CoA (614.8 ± 25.4 pmol/min/mg protein) was lower than with 18:3-CoA, and much lower with 22:5-CoA (63.3 ± 8.3 pmol/min/mg protein) and 22:6-CoA (64.0 ± 5.9 pmol/min/mg protein). These were even lower than the activity observed in the single acyl-CoA fed assays for the relevant acyl-CoAs. Similarly, the specificities of BnLPAAT for 20:5-CoA, 22:5-CoA, and 22:6-CoA were 491.7 ± 23.2, 15.1 ± 4.0, and 5.8 ± 0.8 pmol/min/mg protein, respectively, indicating that the preferences of At- and BnLPAATs for 22:5- and 22:6-CoAs were lower in the presence of 18:3- and 20:5-CoAs. The relative activities of AtLPAAT and BnLPAAT for 22:6-CoA compared with 18:3-CoA were only 9.8% and 0.7%, respectively.

In contrast, MaLPAAT showed the highest specificities for 22:5-CoA and 22:6-CoA (208.2 ± 3.7 and 538.2 ± 6.2 pmol/min/mg protein) among these LPAATs (**Figure 3A**). These activities were also higher than the observations in the single acyl-CoA assays. In addition, MaLPAAT showed 59.8 ± 0.3%, 18.6 ± 0.5%, and 53.3 ± 0.5% relative specificities for 20:5-, 22:5-, and 22:6-CoAs compared with 18:3-CoA in the competition assay, while BnLPAAT showed 60.0 ± 1.2%, 1.8 ± 0.4%, and only 0.7 ± 0.1% specificities for the same acyl-CoAs, respectively, compared with 18:3-CoA. ScLPAAT exhibited much higher specificity for 22:6-CoA (208.2 ± 9.0 pmol/min/mg protein) than AtLPAAT and BnLPAAT, and the relative specificities of ScLPAAT were 19.6 ± 0.7%, 6.9 ± 0.2%, and 36.5 ± 1.5% of the specificity of 18:3-CoA for 20:5-, 22:5-, and 22:6-CoAs, respectively. Although the overall activity of EhLPAAT was lower than MaLPAAT, the relative specificities for 20:5-, 22:5, and 22:6-CoAs were 70.9 ± 7.9%, 41.9 ± 0.8%, and 77.8 ± 2.2% of that for 18:3-CoA, which was much higher than observed for BnLPAAT. MpLPAAT, and LaLPAAT showed very low overall activity, thus the relative activities for 20:5-, 22:5-, and 22:6-CoAs compared with 18:3-CoA were not shown.

We also investigated the preferential transfer of acyl-CoA molecular species by LPAATs from the acyl-CoA pool of multiple species to a different acyl acceptor, 22:6-LPA. We chose 22:6-LPA as a substrate because previous nuclear magnetic resonance (NMR) analysis showed DHA primarily localized to the *sn*-1 and *sn*-3 positions of DHA-Arabidopsis and DHA-camelina TAG (Petrie et al., 2012, 2014), indicating a possible availability of *sn*-1 22:6-LPA in these seeds. In general, the LPAAT activities with 22:6-LPA substrate exhibited a similar pattern of preferences for C20 or C22 LC-PUFA as 18:1-LPA (**Figure 3**), but with some interesting differences. Overall, using the 22:6-LPA substrate resulted in lower LPAAT activities in all the LPAATs investigated, compared to 18:1-LPA substrate. The specificity for 18:3-CoA was decreased in both AtLPAAT and BnLPAAT (319.5 ± 12.8 and 757.5 ± 27.4 pmol/min/mg protein) by 2.1-and 1.1-fold, respectively (**Figure 3B**), compared with the activities of the 18:1-LPA substrate. Moreover, AtLPAAT and BnLPAAT activities with 20:5-CoA were 47.2 ± 7.9 and 117.3 ± 1.2 pmol/min/mg protein, which were, respectively, 13.0-fold and 4.2-fold lower than with 18:1-LPA, whereas the activities with 22:5-CoA were 1.6 ± 2.3 (a dramatic 39.7-fold decrease) and 1.9 ± 0.2 pmol/min/mg protein (8.1-fold decrease), respectively. No activity for 22:6-CoA was observed in either AtLPAAT or BnLPAAT when using 22:6-LPA as an acyl acceptor.

Conversely, MaLPAAT and ScLPAAT showed clear incorporation of 20:5-, 22:5-, and 22:6-CoAs in the PA fraction. MaLPAAT showed 230.9 ± 6.6, 67.7 ± 7.1, and 309.9 ± 31.1 pmol/min/mg protein activities for 20:5-, 22:5-, and 22:6-CoAs, or 38.3, 11.2, and 51.5%, respectively, relative to 18:3-CoA. ScLPAAT showed 38.1 ± 2.6, 20.4 ± 1.6, and 110.3 ± 23.5 pmol/min/mg protein activities for 20:5, 22:5, and 22:6-CoAs, or 8.9, 4.7, and 25.6%, respectively, of the activity with 18:3-CoA. These relative activities were substantially higher than that of BnLPAAT for the same acyl-CoA substrates, in comparison with 18:3-CoA, which were 15.5, 0.2, and 0.0%, respectively.

Similar to their activities with 18:1-LPA, EhLPAAT, LaLPAAT, and MpLPAAT showed low levels of activity for 22:6-LPA. EhLPAAT did not show any specificity for 22:6-CoA in the presence of other acyl-CoAs, but had a high relative activity for 20:5-CoA.

### Enhanced *sn*-2 Distribution of DHA in Transgenic Arabidopsis Seed TAG by MaLPAAT

Based on its substrate preference and overall enzyme activity, we tested MaLPAAT for the possible enrichment of DHA at the TAG *sn*-2 position in seed oil. MaLPAAT, under the control of a seed-specific promoter, was super-transformed into transgenic Arabidopsis seed with pre-existing ability to produce about 10% DHA in the TAG [GA7, (Petrie et al., 2012)]. Fatty acid analysis of mature T_2_ seeds from the super-transformant NY T_1_ and GA7 parent control seeds revealed the presence of higher proportions of DHA in NY than in GA7 parental seeds (**Table 1**). The DHA levels increased 1.35-fold from 11.4% up to 15.5% and the DPA levels in these NY lines were also higher than in GA7. Although the DHA level was increased, the oil content in NY seeds was same as the parent GA7 (data not shown).

**Table 1:** Fatty acid profiles of DHA-Arabidopsis and MaLPAAT super-transformed T_2_ seeds.

Fatty acid	GA7	NY-11	NY-15	NY-16	NY-4	NY-9	NY-30	NY-21	NY-31	NY-20	NY-5	NY-2	NY-17	NY-29	NY-13	NY-14	NY-7	NY-3	NY-23	NY-32	NY-26	NY-27
16:0	9.3	11.5	10.9	10.5	11.5	12.4	11.1	10.6	9.6	10.2	14.6	10.7	9.0	10.2	11.5	9.2	9.3	9.3	8.3	8.5	10.5	9.4
18:0	3.5	3.2	3.3	3.4	3.5	3.7	3.5	3.5	3.0	3.1	4.5	3.3	2.4	3.7	3.4	2.9	2.9	2.8	2.8	3.1	4.3	3.2
18:1n7	2.9	2.5	2.7	4.6	3.2	4.6	4.1	3.8	2.1	3.3	7.7	4.4	2.5	4.3	3.4	2.0	3.8	3.4	1.9	1.9	3.8	5.3
20:0	1.5	2.1	1.9	1.8	2.1	2.3	2.0	2.0	2.0	2.0	2.1	1.9	1.6	1.6	2.2	1.9	2.1	2.2	1.9	1.6	2.6	2.0
20:1n9/n11	12.2	7.6	7.4	8.3	7.9	9.2	9.0	8.6	8.8	8.3	9.9	12.2	8.5	10.4	9.4	9.3	11.2	9.9	11.0	14.3	14.0	13.0
22:1	0.5	0.7	0.5	0.5	0.6	0.6	0.6	0.6	0.8	0.6	0.4	0.7	0.7	0.5	0.8	0.8	0.8	0.8	1.0	0.8	0.9	0.9
Minor	1.7	2.4	2.2	2.3	2.4	3.0	2.3	2.1	1.8	2.4	4.1	2.8	1.9	2.1	3.0	1.8	2.3	2.2	1.6	1.5	2.9	2.4
18:1	7.5	4.5	4.6	6.0	4.6	5.1	5.5	5.6	5.6	5.4	7.0	6.5	5.9	7.6	5.2	6.6	6.7	6.3	7.0	8.0	8.1	7.5
18:2	6.4	7.1	7.0	7.9	7.0	7.1	7.3	8.2	8.5	9.0	6.7	7.6	10.3	8.0	8.3	10.3	9.1	10.3	11.0	9.5	16.2	11.5
18:3n3	33.3	33.3	34.0	30.3	32.7	28.5	30.3	32.0	35.4	33.0	20.8	28.2	35.3	28.9	30.1	34.6	31.5	32.9	34.6	31.8	18.9	28.8
**ω6**
18:3n6	0.5	0.2	0.3	0.3	0.3	0.2	0.3	0.3	0.2	0.2	0.3	0.3	0.2	0.4	0.3	0.2	0.2	0.2	0.2	0.3	0.4	0.2
20:2n6	0.7	0.9	0.8	0.6	0.9	0.8	0.8	0.9	1.1	1.0	0.4	0.7	1.1	0.7	0.9	1.2	0.9	1.1	1.4	1.0	0.9	1.0
20:3n6	0.0	0.0	0.0	0.0	0.0	0.0	0.0	0.0	0.0	0.0	0.0	0.0	0.0	0.0	0.0	0.0	0.0	0.0	0.0	0.0	0.0	0.0
20:4n6	0.0	0.0	0.0	0.0	0.0	0.0	0.0	0.0	0.0	0.0	0.0	0.0	0.0	0.0	0.0	0.0	0.0	0.0	0.0	0.0	0.0	0.0
**ω3**
18:4n3	4.3	3.9	5.1	4.4	4.7	4.1	4.7	4.7	3.9	4.0	5.7	4.3	3.6	4.7	5.0	3.3	3.2	2.7	2.5	3.3	2.4	2.2
20:3n3	1.1	1.9	1.8	1.5	1.8	1.6	1.7	1.6	2.1	1.9	1.0	1.2	1.9	1.1	1.7	1.8	1.6	1.9	2.0	1.1	0.8	1.8
22:3n3	0.1	0.2	0.1	0.1	0.2	0.2	0.2	0.1	0.2	0.2	0.0	0.1	0.2	0.1	0.2	0.2	0.2	0.2	0.2	0.1	0.0	0.3
20:4n3	0.9	0.7	0.8	0.9	0.8	0.7	0.7	0.8	0.9	1.1	0.8	1.1	1.2	0.7	1.5	1.2	1.3	1.3	1.2	1.2	1.9	0.5
22:4n3	0.0	0.0	0.0	0.1	0.0	0.0	0.1	0.1	0.1	0.3	0.0	0.0	0.2	0.1	0.0	0.2	0.2	0.3	0.2	0.1	0.0	0.2
20:5n3	1.6	0.8	1.0	1.1	1.0	1.1	1.1	1.0	0.8	0.8	1.2	1.3	1.0	1.4	1.1	0.9	0.8	0.6	0.7	1.2	1.4	0.6
22:5n3	0.8	1.0	0.9	1.3	0.9	1.2	1.0	0.9	0.8	1.1	1.0	1.1	0.9	1.9	1.0	0.8	1.1	1.0	0.7	0.8	1.4	1.4
22:6n3	11.4	15.5	14.6	14.1	14.1	13.8	13.8	12.6	12.2	12.2	11.7	11.7	11.6	11.6	11.2	11.1	10.8	10.7	9.9	9.8	8.5	7.7


*GA7, parent Arabidopsis line expressing DHA biosynthesis pathway; NY lines, super-transformation lines of GA7 with MaLPAAT.*

The increased level of DHA in NY lines compared to the parent GA7 might be due to a higher preference of MaLPAAT for 22:6-CoA than the endogenous AtLPAAT, leading to the increased transfer of DHA onto the *sn-*2 of the TAG. To confirm this, the purified TAG fraction from the seeds of the primary transgenic lines was digested with *sn*-1/*sn*-3 specific lipase, releasing the free fatty acids from these positions and resulting in *sn*-2 MAG products. GC analysis revealed an increased proportion of DHA in the *sn*-2 MAG of NY seed TAG than in the parent GA7 TAG. The lipase assay showed 37 and 48% of the total DHA in NY11 and NY15, respectively, were present at the *sn-*2 position, compared with 24% in the GA7 parent.

**FIGURE 4:**
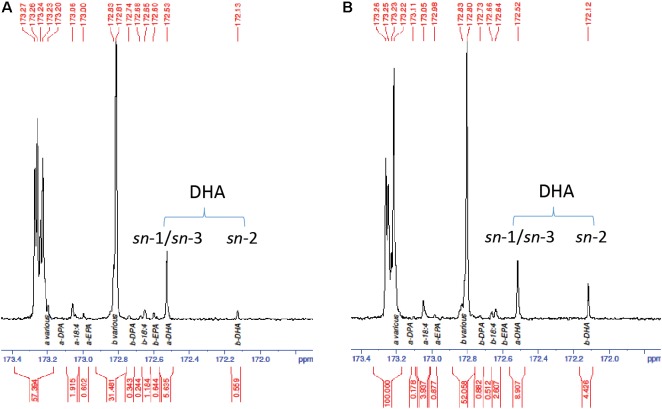
^13^C-NMR spectrum of the carbonyl (C1) region showing the positional distribution of SDA, EPA, DPA, and DHA in transgenic Arabidopsis seed TAG. **(A)** DHA-Arabidopsis parent GA7 seeds. **(B)** T_4_ seeds of NY15, super-transformed with MaLPAAT in GA7. *a* and *b* denotes the fatty acids at *sn*-1/*sn*-3 and *sn*-2 positions of TAG, respectively.

The increased DHA levels in seed oil and at the TAG *sn*-2 position were further confirmed by GC analysis in the homozygous progeny. Line NY15 that showed a single T-DNA insertion based on *NptII* segregation and higher DHA in T_2_ seeds was chosen to follow up in the next generations. The kanamycin resistant T_2_ seedlings were planted in the glasshouse, along with parent GA7 control. The existence of *MaLPAAT* transgene of these progenies was confirmed by *MaLPAAT* specific PCR. DHA in NY15 T_3_ seeds from these progenies had a 1.14- to 1.23-fold increase compared to GA7 seeds (**Table 2**), suggesting the DHA increase was associated with MaLPAAT. Preliminary acyl-CoA substrate competition assay of Arabidopsis NY15 developing seed microsome with 18:1-LPA showed similar activity on 20:5 or 22:6 incorporation in PA product compared to 18:3 (**Supplementary Figure S1**). DHA positional distribution on the TAG was analyzed with NMR in pooled T_4_ seeds from the homozygous NY15. The NMR spectrum showed that the DHA was predominantly located at the *sn*-1/*sn*-3 (α)-positions of GA7 Arabidopsis seed TAG (91% of total) and only 9% of it resided at the *sn*-2 (β)-position (**Figure 4A**). In contrast, 33.2% of the DHA was located at the *sn*-2 position in NY15 seed TAG, leaving 66.8% at the *sn*-1/*sn*-3 positions (**Figure 4B**). These results further confirmed the ability of MaLPAAT to enrich DHA at the TAG *sn*-2 position in transgenic Arabidopsis seed.

**Table 2:** Fatty acid profiles of DHA-Arabidopsis and MaLPAAT super-transformed T_3_ seeds.

Fatty acid	GA7 T_5_ (*n* = 4)	NY15.5.x T_3_ (*n* = 4)	NY15.6.x T_3_ (*n* = 5)
16:0	8.6 ± 0.2	9.9 ± 0.6	9.8 ± 0.1
18:0	2.8 ± 0.0	2.8 ± 0.1	2.7 ± 0.0
18:1n7	1.9 ± 0.1	2.1 ± 0.6	1.7 ± 0.1
20:0	1.3 ± 0.0	1.7 ± 0.2	1.9 ± 0.1
20:1n9/n11	14.3 ± 0.5	7.7 ± 0.6	8.0 ± 0.3
22:1	0.8 ± 0.0	0.7 ± 0.1	0.9 ± 0.1
Minor	1.3 ± 0.1	1.6 ± 0.3	1.5 ± 0.1
18:1	9.4 ± 0.3	5.6 ± 0.4	5.3 ± 0.3
18:2	9.2 ± 0.3	9.3 ± 1.5	9.2 ± 0.5
18:3	33.0 ± 0.3	38.1 ± 1.2	39.6 ± 0.5
**ω6**
18:3n6	0.3 ± 0.0	0.2 ± 0.0	0.1 ± 0.0
20:2n6	0.9 ± 0.0	1.2 ± 0.2	1.4 ± 0.1
20:3n6	0.1 ± 0.1	0.2 ± 0.0	0.2 ± 0.1
20:4n6	0.0 ± 0.0	0.0 ± 0.0	0.0 ± 0.0
**ω3**
18:4n3	2.7 ± 0.3	3.0 ± 0.3	2.7 ± 0.3
20:3n3	1.1 ± 0.0	2.2 ± 0.3	2.6 ± 0.2
22:3n3	0.1 ± 0.0	0.2 ± 0.0	0.3 ± 0.0
20:4n3	1.1 ± 0.1	0.8 ± 0.0	0.7 ± 0.1
22:4n3	0.0 ± 0.0	0.2 ± 0.0	0.2 ± 0.0
20:5n3	1.1 ± 0.1	0.6 ± 0.3	0.4 ± 0.1
22:5n3	0.8 ± 0.1	0.6 ± 0.2	0.4 ± 0.1
22:6n3	9.3 ± 0.3	11.5 ± 1.6	10.6 ± 0.7


*GA7, parent Arabidopsis line expressing DHA biosynthesis pathway; NY progenies, super-transformation lines of GA7 with MaLPAAT.*

### LC-MS Analysis of DHA Containing Neutral Lipids

**FIGURE 5:**
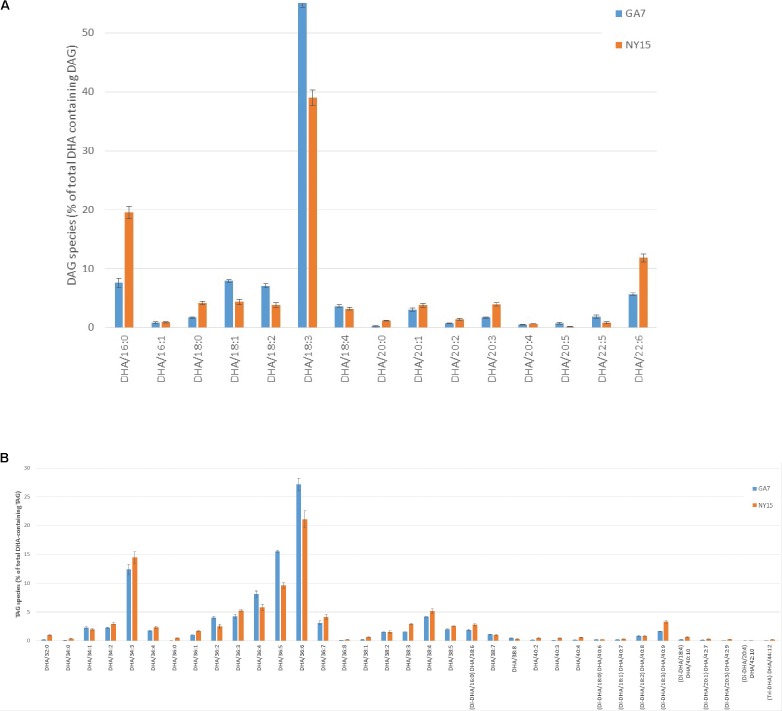
Distribution of DHA-containing DAG and TAG species in Arabidopsis seeds. **(A)** DHA-containing DAG species. **(B)** DHA-containing TAG species. GA7, lipids from the DHA-Arabidopsis parent plant; NY15, lipids from the super-transformed lines with MaLPAAT in GA7. DHA species with very low percentage levels are not listed.

Because AtLPAAT and BnLPAAT had very low acylation activity of 22:6-CoA onto 22:6-LPA compared with MaLPAAT (**Figure 3B**), we hypothesized that overexpression of MaLPAAT in Arabidopsis or *B. napus* might result in higher levels of TAGs containing multiple 22:6 in the seed. Indeed, LC-MS analysis of total lipids from the homozygous NY15 T_4_ seeds showed increased di-DHA DAG, di-DHA and tri-DHA TAGs compared with its GA7 parental line (**Figure 5**). Di-DHA DAG increased from 5.65% of DHA-containing DAGs in GA7 seed to 11.82% of DHA-containing DAGs in NY15. Similarly, di-DHA TAG increased from 5.47% of total DHA-containing TAG in GA7 to 9.3% NY15, and tri-DHA TAG increased from 0.08% of DHA-containing TAGs in GA7 seed to 0.22% of DHA-containing TAGs in NY15 seeds. These results further supported the higher preference of MaLPAAT to acylate DHA onto LPA substrates.

## Discussion

Our previous metabolic engineering of the DHA biosynthesis pathway in Arabidopsis or camelina seeds led to 10–15% DHA accumulation (Petrie et al., 2012, 2014), which are similar to levels in fish oil. The distribution of the DHA was mainly at the TAG *sn-*1/*sn-*3 positions, suggesting that Arabidopsis or camelina enzymes might have a low preference for DHA compared to other fatty acids for acylation of the *sn-*2 position of the glycerol backbone, mediated by LPAAT. These observations triggered the comparison of LC-PUFA preference of a range of LPAATs, as well as the search for LC-PUFA-specific LPAAT enzymes that could increase both the amount of DHA and the accumulation of DHA at the TAG *sn-*2 position in these engineered plant seed oils.

Lysophosphatidic acid acyltransferases have been shown to have differential acyl-substrate preferences. In considering the target LPAATs to be tested in this study, we chose ScLPAAT from yeast and LaLPAAT from meadowfoam, which have preferences for long chain monounsaturated fatty acid (Cao et al., 1990), MaLPAAT from *M. alpina*, MpLPAAT from microalga *M. pusilla*, and EhLPAAT from phytoplankton *E. huxleyi*, which accumulate high amounts of LC-PUFA arachidonic acid (20:4ω6, ARA) or DHA. These were compared with AtLPAAT or BnLPAAT from Arabidopsis or *B. napus*, which lack ω3 LC-PUFA in native seed oil. The substrate preference of some LPAATs has been tested previously by expressing them in human prostate tumor cell lines (Hollenback et al., 2006). In this study, we used transient leaf expression systems to test their preference for ω3 LC-PUFA substrates in plants.

Our results showed that AtLPAAT, BnLPAAT, MaLPAAT, ScLPAAT, and EhLPAAT were active when expressed in *N. benthamiana* leaves, while LaLPAAT and MpLPAAT had low activities in this model. A comparison of LPAAT activity for ALA or downstream LC-PUFA products in the DHA biosynthesis pathway, including EPA, DPA, and DHA, indicated that all these tested LPAAs showed high activities favored 18:3-CoA. It was not surprising that LPAATs isolated from organisms that synthesize PUFAs should inherently have high affinity 18:3 as this fatty acid is consistently placed preferentially at *sn*-2 position of TAG. The results suggested that the Arabidopsis AtLPAAT and *B. napus* BnLPAAT had a relatively high activity for EPA, but indeed low activity for DHA. In contrast, MaLPAAT showed higher activity for DHA, although the highest activity among those four acyl-CoA substrates was for 18:3-CoA. In the real cell environment, however, the acyl-CoA pool is more complicated than in a single acyl-CoA environment. Thus the enzymatic assay with multiple acyl-CoAs provided an understanding of the substrate competition for the enzymes. Although AtLPAAT and BnLPAAT showed very low activity for DHA in a mixture of 18:3-, 20:5-, 22:5-, and 22:6-CoA substrates, MaLPAAT showed strong activity for DHA. MaLPAAT was thus predicted as the best candidate enzyme, among those tested, for enhancing DHA distribution onto the TAG *sn-*2 position in Arabidopsis or canola. It was worth to note that the activity of MaLPAAT for 18:3-CoA in the multiple acyl-CoA assay was higher than with single 18:3-CoA substrate, suggesting the possible discrepancy in activities on certain substrates, when multiple acyl-CoA substrates were available. However, the relative activity for 22:6-CoA to 18:3-CoA in the competition assay was similar to that relative activity in single acyl-CoA substrate assay. It should also point out that the higher enzyme activity might also be caused by *N. benthamiana* leaf variation between batches for transient expression. MaLPAAT also showed the highest activity for DPA among seven tested enzymes in the competition assay.

Glycerol-3-phopsphate acyltransferase (GPAT) catalyzes the acylation of *sn-1* position of the glycerol backbone to make LPA. For a maximal increase of DHA at all three positions of TAG, accumulation of DHA at the *sn-1* position, or the production of 22:6-LPA, is desired. The resulting 22:6-LPA then can be used for the production of di-DHA PA (22:6/22:6-PA), and subsequently for di-DHA DAG, tri-DHA TAG. Therefore, we also analyzed the substrate preference of LPAATs when 22:6-LPA was provided as the acyl receiver. Again, MaLPAAT showed much higher activity for 22:6-CoA than other LPAATs. These led to the further experiment to overexpress MaLPAAT in the Arabidopsis line previously engineered for DHA production. The overexpression of MaLPAAT indeed resulted in the moderate increase of total DHA levels. The existence of relatively high level of 18:3 in DHA producing Arabidopsis seed might compete with 22:6, thus limiting the overall DHA increase in seed. There might be also the competition from the endogenous LPAAT which prefers to 18:3 instead of 22:6. Additionally, MaLPAAT itself has nearly twofold higher activity for 18:3 than for 22:6. However, the overexpression of MaLPAAT did result in the increased distribution of DHA to the TAG *sn-*2. The enrichment of DHA at *sn*-2 by MaLPAAT was further supported by LC-MS analysis showing an increased ratio of multiple DHA in DAG or TAG among all DHA-containing species. These results demonstrated that the expression of the DHA biosynthesis pathway combined with MaLPAAT could enhance the DHA levels at the TAG *sn-*2 position as well as in total seed oil.

Our LPAAT activity assay also confirmed that LPAATs from different sources have variable activities for different LC-PUFAs. Metabolic engineering of other LC-PUFAs might need different enzymes in different crops. For example, Arabidopsis or *B. napus* LPAATs had a relatively high activity for EPA when 18:1-LPA was used as the acyl receiver, suggesting that Arabidopsis or *B. napus* might be suitable for EPA production without additional LPAAT. However, Arabidopsis or *B. napus* have a diversity of LPAAT isoenzymes, which have been shown to have different acyl preference, or additive effect (Roscoe, 2005; Maisonneuve et al., 2010). BnLPAAT used in this work, previously designated BAT1.5, has been shown to have a higher preference to 18:1-CoA than to 16:0-CoA in *Escherichia coli*, with higher LPAAT activity than another isoenzyme BAT1.13 (Maisonneuve et al., 2010). Our study showed that this BnLPAAT had very low activity in loading 22:6-CoA onto 22:6-LPA. This suggested that other LPAAT with higher substrate preference for 22:6-CoA might be needed in engineered *B. napus* to maximize the DHA level in seed oil. Also, there may be a need to silence the endogenous multiple LPAAT activity to remove competition, thus allowing more DHA to be acylated onto LPA.

In summary, our results demonstrated that *M. alpina* MaLPAAT had a higher activity for 22:6-CoA. Co-expression of MaLPAAT with the DHA biosynthesis pathway in Arabidopsis led to the enrichment of DHA at the *sn-*2 position of the TAG, leading to higher DHA production.

## Author Contributions

PS, SS, JP, and X-RZ designed the research. PS, DH, RM, MT, and X-RZ performed the research. PS, DH, SS, JP, and X-RZ analyzed the data. PS, SS, and X-RZ wrote the paper.

## Conflict of Interest Statement

The authors declare that the research was conducted in the absence of any commercial or financial relationships that could be construed as a potential conflict of interest.

## Supplementary Material

FIGURE S1 Competition among acyl-CoA molecular species for acylation into 18:1-LPA by microsomal proteins of Arabidopsis developing seeds. Microsomal proteins were prepared from the developing siliques of NY15 that was supertransformed with *MaLPAAT*. 300 μg of microsomal proteins were used with 500 μM of 18:1-LPA, 100 μM of 18:3-, 20:5-, and 22:6-CoA each, in total of 200 μL of 0.1 M KPO_4_ buffer pH 7.2 with 50 μg of fatty acid-free BSA, for 1 h. PA fraction was separated by TLC fractionated, followed by GC analysis to quantify the PA from different acyl-CoAs in the mixture.

## Abbreviations

CoA: coenzyme A
DAG: diacylglycerol
DHA: docosahexaenoic acid
DPA: docosapentaenoic acid
EPA: eicosapentaenoic acid
GC: gas chromatography
LC-PUFA: long chain polyunsaturated fatty acid
LPA: lysophosphatidic acid
LPAAT: lysophosphatidic acid acyltransferase
MAG: monoacylglycerol
MRM: multiple reaction monitoring
PA: phosphatidic acid
TAG: triacylglycerol
TLC: thin layer chromatography
