# Supplementary figures and images for: Increased DHA Production in Seed Oil Using a Selective Lysophosphatidic Acid Acyltransferase
Source: Front Plant Sci. 2018 Aug 22;9:1234. doi: 10.3389/fpls.2018.01234 (PMC6113368; doi:10.3389/fpls.2018.01234)

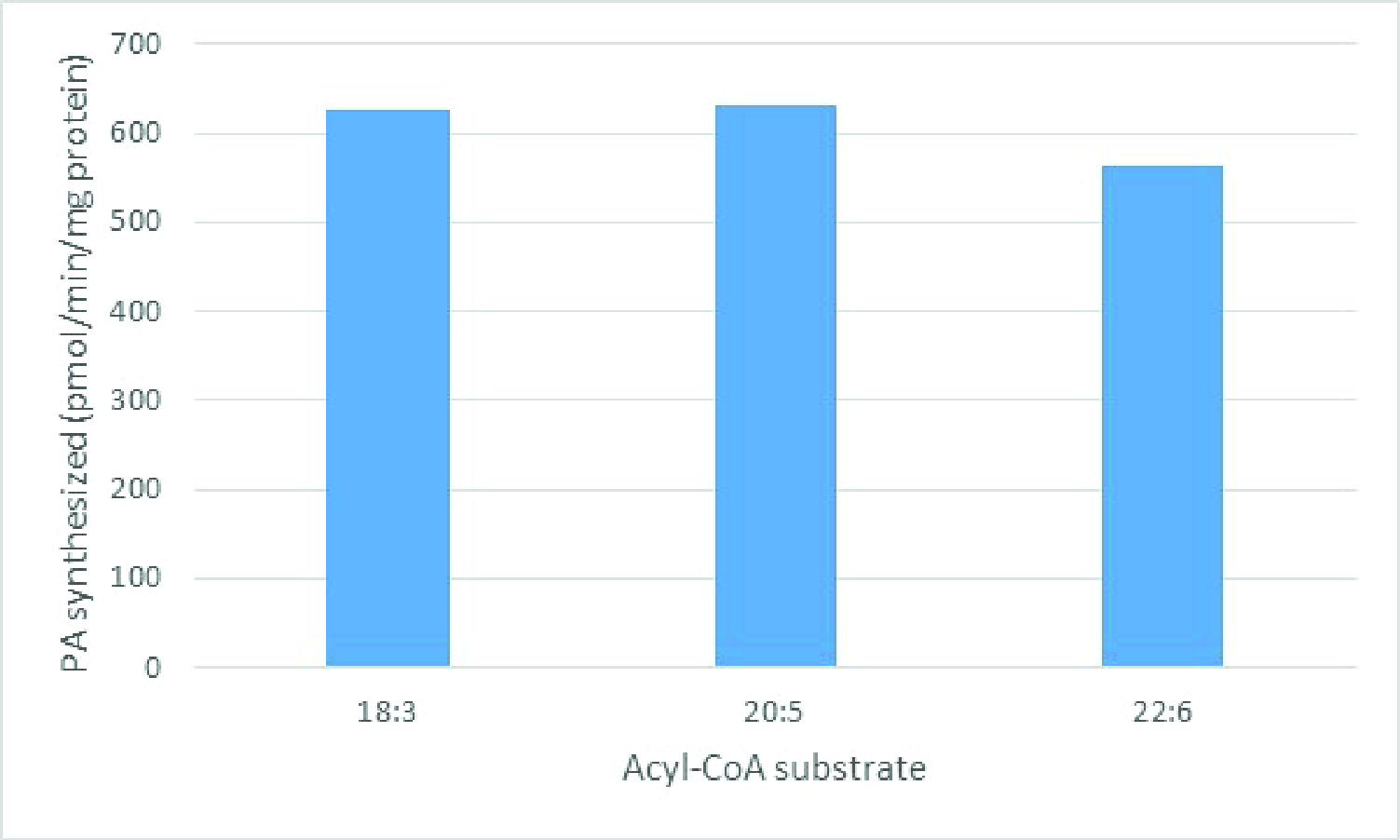

Supplement: FIGURE S1 — Competition among acyl-CoA molecular species for acylation into 18:1-LPA by microsomal proteins of Arabidopsis developing seeds. Microsomal proteins were prepared from the developing siliques of NY15 that was supertransformed with MaLPAAT. 300 μg of microsomal proteins were used with 500 μM of 18:1-LPA, 100 μM of 18:3-, 20:5-, and 22:6-CoA each, in total of 200 μL of 0.1 M KPO4 buffer pH 7.2 with 50 μg of fatty acid-free BSA, for 1 h. PA fraction was separated by TLC fractionated, followed by GC analysis to quantify the PA from different acyl-CoAs in the mixture. [file Image_1.JPEG]
